# Copper Ion Removal Using a Waste-Plastic-Derived Hydrogel Adsorbent Prepared via Microwave-Assisted PET Aminolysis

**DOI:** 10.3390/gels9110874

**Published:** 2023-11-03

**Authors:** Kayee Chan, Masami Kawai, Mina Yamake, Anatoly Zinchenko

**Affiliations:** 1 Graduate School of Environmental Studies, Nagoya University, Furo-cho, Chikusa-ku, Nagoya 464-8601, Japan; 2 Gifu High School, 3-1, Onawaba, Gifu 500-8889, Japan; 3 Gifu Kita High School, 1841-11, Noritake, Gifu 502-0931, Japan

**Keywords:** plastic recycling, microwave-assisted synthesis, PET, aminolysis, hydrogel, metal ion adsorption, copper

## Abstract

Despite the tremendous progress in the development of functional materials from plastic waste to promote its recycling, only a few examples of hydrogel materials from plastic waste were reported. In this study, microwave-assisted depolymerization of waste PET plastic using polyamine was performed to prepare short aminophthalamide oligomers followed by chemically cross-linking into a hydrogel material. Catalyst-free microwave-assisted aminolysis of PET was completed within 30–40 s, demonstrating high efficiency of the depolymerization reaction. Subsequent epoxy cross-linking of the oligomers yielded a hydrogel with a swelling degree of ca. 92.1 times in pure water. The application of the obtained hydrogel for the removal of copper ions (Cu^2+^) from water was demonstrated. Efficient complexation of NH_2_ groups of the hydrogel with Cu^2+^ resulted in high adsorption capacities of the hydrogel material toward Cu^2+^ removal, which were the highest at neutral pHs and reached ca. 213 mg/g. The proposed type of environmental material is beneficial owing to its waste-derived nature and functionality that can be applied for the high-efficiency removal of a broad scope of known environmental pollutants.

## 1. Introduction

Sustainable use of materials implies minimization of non-renewable resource usage and the extension of materials’ lifetime by promoting their circulation via recycling, upcycling, downcycling, and other approaches. The contemporary design of sustainable adsorbents for wastewater treatment takes advantage of using either renewable biomass resources [1,2] or, more frequently, various types of waste [3] that have been extensively studied over the past decade. In particular, the utilization of waste plastics for the preparation of adsorbents was increasingly considered, and various functional adsorbents for the removal of dyes [4], removal of toxic metals [5], sequestration of CO_2_ [6], and other applications were designed.

On the other hand, hydrogels, particularly those prepared from biomass, represent a well-established class of adsorbents that demonstrate useful characteristics [7,8,9,10] due to their non-invasive character, the absence of secondary contamination during water treatment, and high adsorption capacities thanks to the accessibility of the adsorbate molecules to the binding sites on the macromolecular chains of hydrogels. However, the design and preparation of waste-plastic-derived hydrogels is still a challenge. The intrinsically hydrophobic nature of the fossil-derived starting materials and the associated difficulties in the chemical transformation of waste plastics to water-soluble precursors with a controlled molecular architecture are the major difficulties to the construction of a water-soluble polymer network of a hydrogel.

In our recent works, we successfully prepared a hydrogel from waste PET via aminolytic depolymerization to oligomeric “building blocks” of terephthalamides and their cross-linking [11,12]. The cationic character of the hydrogel material was exploited for the adsorption of anionic species such as industrial dyes [11], whereas abundant amines of the hydrogel are also well known as ligands for transition metal ions, some of which (Cd^2+^, Cu^2+^, Pb^2+^, etc.) are toxic environmental pollutants [1]. In this regard, a vast number of NH_2_-containing and NH_2_-functionalized adsorbents have been elaborated, and their application for the removal of metal ions has been shown [13]. Herein, we report an optimized, microwave-assisted, PET depolymerization protocol via aminolysis to prepare oligomeric terephthalamides for the consequent synthesis of a hydrogel. Consequently, we demonstrate for the first time a successful application of PET-derived hydrogel for the removal of Cu^2+^ ions from aqueous solutions with a high adsorption capacity of 213 mg/g at neutral pH. In our study, the adsorption properties of the developed hydrogel adsorbent outperformed most earlier-reported amine-containing adsorbents.

## 2. Results and Discussion

### 2.1. Formation and Characterization of Hydrogel Adsorbent via Microwave-Assisted Aminolysis of PET

**Figure 1:**
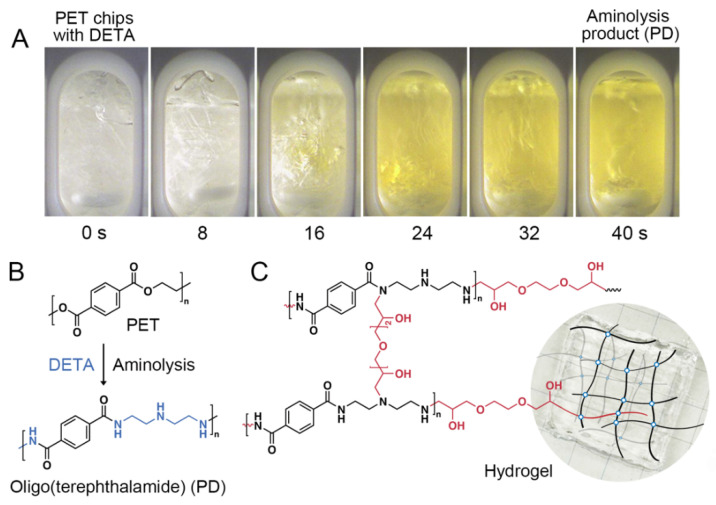
(**A**) Time snapshots of PET and DETA reaction mixture in the microwave reactor vessel under continuous stirring. The reaction time in seconds is shown under each image. (**B**) Schematic illustration of the structure of PET depolymerization to PD by diethylenetriamine. (**C**) The photograph, schematic illustration of the structure, and the chemical structure of the cross-linked hydrogel.

Aminolytic depolymerization of PET through a reaction with diethylenetriamine (DETA) (Figure 1A,B) was a modified procedure of [11], but the reaction was performed in a microwave reactor. Under microwave heating, a reaction temperature of 200 °C was established within 10 s, with no notable increase in the autogenous pressure in the reaction vessel (Figure S1). During the reaction, PET chips were gradually dissolved while the solution color turned yellow (Figure 1A), which corresponded to the formation of terephthalamides from PET. Using the microwave approach, the reaction of the complete PET depolymerization took only 30–40 s in contrast to ca. 5–10 min depolymerization time using conventional organic synthesis in the presence of zinc acetate as a catalyst [11]. The major product of PET depolymerization obtained in a triple-excess DETA is an oligomeric terephthalamide, which is hereafter referred to as PD for PET/DETA.

**Figure 2:**
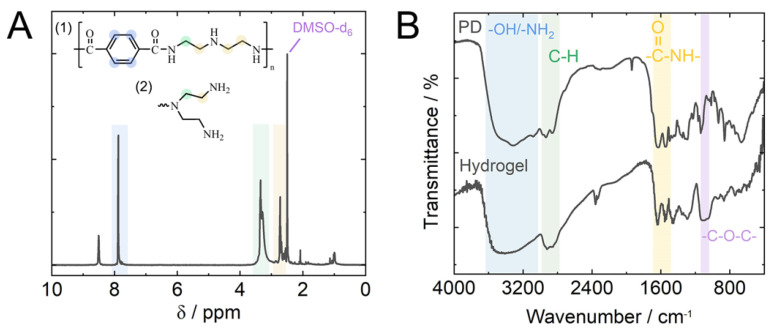
(**A**) ^1^H NMR spectrum of PET aminolysis product (PD). The correspondence of NMR signals and protons in the PD structure is shown by specific colours. (**B**) FTIR spectra of the PD and freeze–dried hydrogel.

The PET depolymerization products were precipitated into acetone, and the solid fraction was dried in an oven at 75 °C, and characterized via FTIR and NMR spectroscopies. The essential characteristics of the aminolysis product were the same as those reported for the conventional organic depolymerization reaction “in flask” [11]. The primary and secondary amines of DETA can attack the ester bonds of PET, resulting in the two possible structures of PET aminolysis products. Due to the higher reactivity of primary amines, the structure (1) of the PET aminolysis product shown in Figure 2A is the main reaction product. As shown in the ^1^H NMR spectrum of PD, the peak at around 7.8 ppm corresponds to the aromatic protons. The peaks at 3.3–3.2 ppm, which overlapped with the peak of water at 3.3 ppm, correspond to the protons of methylene groups adjacent to the amide groups. The peaks at around 2.7–2.5 ppm were assigned to the protons of methylene groups adjacent to the amine groups. These results indicated the formation of amide bonds between terephthalic acid residues and the amines of DETA. In addition, the FTIR spectrum of PD was consistent with the NMR data, indicating the formation of amide groups by the appearance of C=O stretching band at 1636 cm^−1^ and N-H bending band at 1545 cm^−1^ (Figure 2B). The peaks at 2955–2815 cm^−1^ in the FTIR spectrum of PD indicated the C-H stretch of aromatics of terephthalic residues and alkyl of amine (Figure 2B). A comparison of FTIR and NMR spectra of microwave reaction products with the organic reaction products reported earlier [11] showed that the microwave approach enhances the reaction efficiency in terms of lower consumed energy, no catalyst, and short reaction time, but has no significant effect on the structure and composition of PET decomposition products.

Hydrogel material was prepared by cross-linking of the PD with ethylene glycol glycidyl ether (EGDE) (Figure 1C) under moderate heating (50 °C). The swelling degree of the hydrogel that was repeatedly washed and equilibrated against pure water to reach a constant volume was ca. 92.1 under neutral pH conditions.

**Figure 3:**
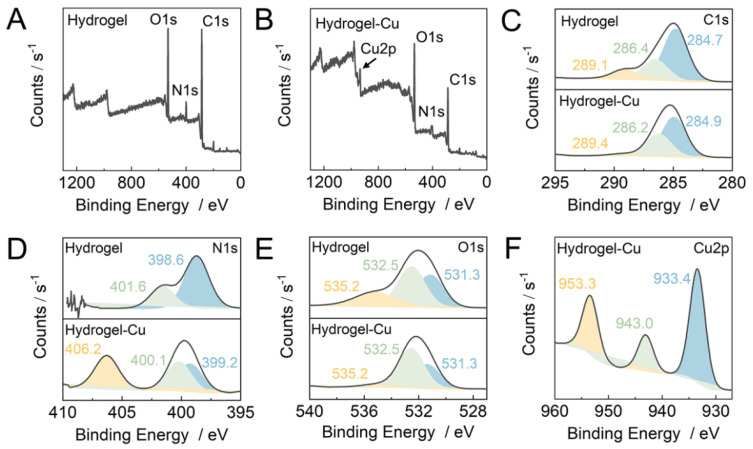
(**A**,**B**) XPS survey spectrum of hydrogel before (**A**) and after (**B**) Cu^2+^ adsorption. (**C**–**F**) High–resolution C1s (**C**), N1s (**D**), O1s (**E**) of the hydrogel before and after Cu^2+^ adsorption, and Cu2p spectra of the hydrogel after Cu^2+^ adsorption (**F**).

The successful cross-linking of the PD was confirmed by the appearance of the -C-O-C- stretching peak at 1089 cm^−1^ in the FTIR spectrum of the hydrogel (Figure 2B). Further information about the chemical composition and bonding states of atoms of the hydrogel material was obtained by XPS analysis (Figure 3). The XPS survey spectrum of hydrogel clearly showed the pronounced signals of C1s (70.2%) at around 285.9 eV, N1s (7.6%) at around 399.6 eV, and O1s (22.2%) at around 532.3 eV (Figure 3A). The carbon bonding states were confirmed by a high-resolution C1s spectrum that contained the peaks of C-C/C=C bonds at 284.7 eV, C-N/C-O bonds at 286.4 eV, and C=O bonds at 289.1 eV (Figure 3C). In addition, a high-resolution N1s spectrum (Figure 3D) indicated two nitrogen bonding states of -NH_2_ at 398.6 eV and O=C-N at 401.6 eV [14], while three oxygen bonding states of C=O at 531.3 eV, -C-O-C- at 532.5 eV, and C-OH at 535.2 eV were found in a high-resolution O1s spectrum (Figure 3E) [15]. The results from the XPS analysis were in good agreement with those of the FTIR.

### 2.2. Removal of Cu^2+^ Ions from Aqueous Solution by the PET-Derived Hydrogel

**Figure 4:**
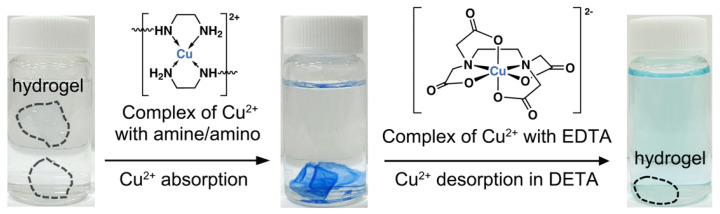
Photographic images of the PET-derived hydrogel before (**left**) and after Cu^2+^ adsorption (**middle**), and after Cu^2+^ desorption in a 0.1 M EDTA solution (**right**). The plausible structures of the Cu^2+^ coordination complexes with two ethylenediamine moieties of the hydrogel and EDTA are shown above the arrows of the adsorption and desorption processes.

Next, the obtained hydrogel was used for Cu^2+^ adsorption experiments. Soaking of the hydrogel in 127 mg/L Cu(NO_3_)_2_ solution resulted in the appearance of the intense blue colour of the hydrogel (Figure 4), indicating the efficient Cu^2+^ uptake and complex formation. The uptake mechanism is the coordination of Cu^2+^ with the amine/amino groups of the hydrogel into a complex having the plausible structure shown in Figure 4 above the adsorption process arrow. The high stability of the Cu^2+^ complexes with the amines of the hydrogel is particularly favoured by the presence of ethylene diamine fragments from DETA that form stable coordination complexes with Cu^2+^ [16]. Adsorption of Cu^2+^ was accompanied by a moderate swelling of the hydrogel that can be attributed to the additional contribution of the electrostatic repulsions between like-charged Cu^2+^ ions bound to the neutral amines in the hydrogel matrix.

**Figure 5:**
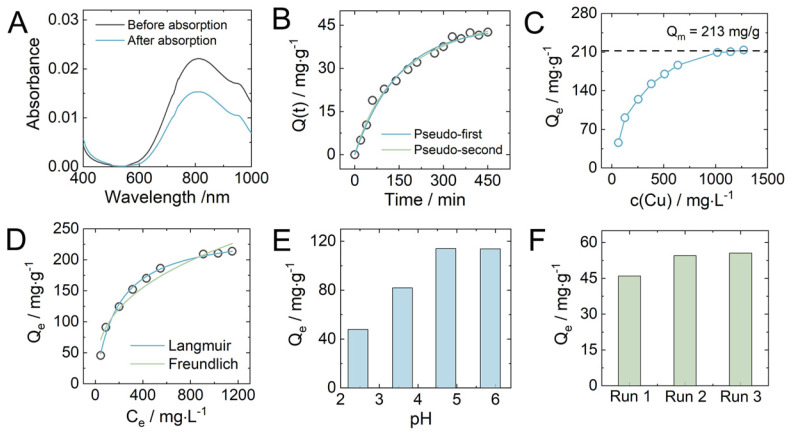
(**A**) UV–vis spectra of Cu^2+^ before and after adsorption by ca. 0.13 g of hydrogel from 127 mg/L Cu^2+^ solution. (**B**) Kinetics of Cu^2+^ adsorption by ca. 1 g of hydrogel from a 127 mg/L Cu^2+^ solution. The non–linear fit of the adsorption kinetics data using the pseudo–first–order and pseudo–second–order kinetics models. (**C**) Adsorption capacities of ca. 0.15 g of hydrogel for Cu^2+^ adsorption at different concentrations of Cu^2+^ in solution. (**D**) Non–linear fit of the adsorption data using the Langmuir and Freundlich isotherm models. (**E**) Dependence of adsorption capacities of the hydrogel for Cu^2+^ adsorption on the pH of Cu^2+^ solution. (**F**) Adsorption capacities of ca. 1 g of hydrogel for Cu^2+^ adsorption from a solution with a concentration of 127 mg/L during repeating adsorption/desorption experiments by using the same hydrogel adsorbent.

Batch adsorption experiments were carried out to study Cu^2+^ adsorption characteristics of PET-derived hydrogel in aqueous solutions. The concentration of Cu^2+^ was measured spectroscopically at the absorbance wavelength of 810 nm. Stirring the hydrogel in an aqueous Cu^2+^ solution resulted in a progressive decrease in the absorbance of Cu^2+^ remaining in the solution above the hydrogel (Figure 5A). Time-dependent changes in Cu^2+^ concentration showed a gradual uptake of Cu^2+^ by the hydrogel, that reached saturation within several hours (Figure 5B). The obtained kinetic data were further fitted to pseudo-first- and pseudo-second-order adsorption kinetics models, which indicate mechanical and chemical adsorption mechanisms [17], respectively. A higher *R*^2^ value (0.9909) (Table 1) for the pseudo-seconds kinetics fitting indicates the chemical adsorption mechanism of Cu^2+^ and correlates with the adsorption kinetics of anionic dye, Congo Red, by the same type of hydrogel reported earlier [11].

The effect of the initial Cu^2+^ concentrations on the adsorption capacities of hydrogel is shown in Figure 5C. The maximum adsorption capacity of the hydrogel toward Cu^2+^ was found to be 213 mg/g. To get insight into the Cu^2+^ adsorption mechanism by the hydrogel, adsorption isotherms were measured at room temperature and fitted by the Langmuir and Freundlich isotherm models, which indicate the monolayer and multilayer adsorption mechanism [18], respectively (Figure 5D). The better fitting results (*R*^2^ = 0.9930) using the Langmuir model indicated the monolayer adsorption mechanism. The maximum adsorption capacity of the hydrogel calculated by the Langmuir isotherm model was found to be 248 mg/g (Table 1), which is slightly higher than the experimental Q_m_ of 213 mg/g, also suggesting a good match of the Langmuir isotherm model for the adsorption process of the hydrogel for Cu^2+^. The best-fitting adsorption model for Cu^2+^ adsorption was again the same as was reported earlier for the hydrogel adsorption of the Congo Red cationic dye [11]. However, the adsorption constant of the hydrogel (*K*_L_ = 0.0054) for Cu^2+^ ions was two orders of magnitude lower than the cationic dye Congo Red, indicating significantly a weaker interaction of the hydrogel with Cu^2+^ compared to organic molecules of Congo Red. This difference can be attributed to additional hydrophobic interactions of Congo Red with the hydrogel macromolecules containing the hydrophobic regions of terephthalic acid residues.

The effect of the pH of the Cu^2+^ solution on the adsorption capacity of hydrogel was studied within the pH range of 2–6, due to the formation of metal hydroxide precipitates of Cu^2+^ at a higher pH [19]. As shown in Figure 5E, no significant change in Q_e_ within the pH range of 4–6 was observed, while with a decrease in pH to 2.4, the adsorption capacity of the hydrogel was greatly decreased ca. 3-fold. Under the lower pH values of the Cu^2+^ solution, the protonation of the amine groups in the hydrogel occurred to form -NH_3_^+^ species. The electrostatic repulsion between Cu^2+^ ions and -NH_3_^+^ species resulted in a decrease in Q_e_.

**Figure 6:**
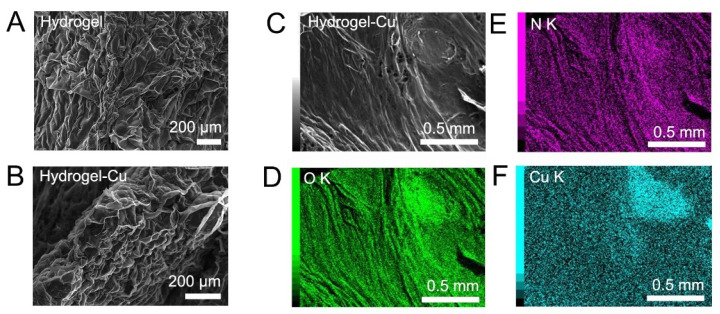
(**A**,**B**) SEM images of the freeze-dried hydrogel before (**A**) and after (**B**) Cu^2+^ adsorption. (**C**,**F**) Elemental mapping of O (**D**), N (**E**), and Cu (**F**) elements in the hydrogel after Cu^2+^ adsorption (**C**).

SEM images of the freeze-dried hydrogel before and after Cu^2+^ adsorption are shown in Figure 6A,B, together with the mapping of N, O, and Cu elements (Figure 6C–F). The surface morphology of both hydrogels showed a moderate porosity and no significant difference in hydrogels before and after Cu^2+^ adsorption was observed. Elemental mapping indicated the coincidence of N, O, and Cu elemental densities, suggesting homogenous Cu^2+^ uptake by the polymeric matrix of the adsorbent.

Furthermore, the Cu^2+^ adsorption mechanism by the hydrogel was studied via XPS analysis (Figure 3). The XPS survey spectrum of hydrogel after Cu^2+^ adsorption (hydrogel–Cu) showed the pronounced signals of C1s (63.7%) at around 287.5 eV, N1s (3.3%) at around 401.3 eV, O1s (31.0%) at around 534.0 eV, and Cu2p (1.98%) at around 934.1 eV (Figure 3B). While the theoretical N/Cu ratio based on the structure of the complex illustrated in Figure 4 should be 4, the measured N/Cu ratio was ca. 1.6, which might be due to the consumption of a part of amines for cross-linking, as well as structural constraints in the hydrogel for the coordination of Cu^2+^ with two binding sites. Compared with XPS data of hydrogel, the carbon and oxygen bonding states of hydrogel–Cu were comparable with the hydrogel (Figure 3C,E). However, the obvious shifts in characteristic peaks of the amine at 399.2 eV and amide at 400.1 eV were observed in a high-resolution N1s spectrum of hydrogel–Cu (Figure 3D), indicating that the amine/amide groups participated in the adsorption reaction with Cu^2+^ ions. In addition, a new peak at 406.2 eV was assigned to the Cu-N, further suggesting the coordination chelation between amine/amino and Cu^2+^ [20]. As shown in Figure 3F, the peaks at 933.4 eV and 953.3 eV were assigned to Cu2p_3/2_ and Cu2p_1/2_, respectively, while an obvious satellite peak at 943.0 eV was also observed in a high-resolution Cu2p spectrum [21].

To study the reusability of the hydrogel adsorbent, a conventional strong metal ion ligand, ethylenediamine tetraacetate (EDTA), was used. The structure of the complex between Cu^2+^ ion and EDTA is shown in Figure 4 above the desorption arrow. After placement of the hydrogel containing Cu^2+^ into a solution of EDTA and gentle mixing, the color of the hydrogel vanished within ca. 120 min, while the outer solution turned light blue indicating successful desorption of Cu^2+^ ion from the hydrogel (Figure 4). Shrinking of the hydrogel was also observed, indicating the transition of the hydrogel to its original state with a higher swelling degree. As shown in Figure 5F, the adsorption capacities of the hydrogel in three consecutive Cu^2+^ adsorption cycles were calculated to be 46.0 mg/g, 54.5 mg/g, and 55.6 mg/g, respectively, suggesting that the hydrogel is highly reusable. A slight increase in the adsorption capacity was due to the shrinking of the hydrogel after treatment with EDTA, resulting in a slight difference in the initial Cu^2+^ concentration between the first cycle and second/third cycles.

The adsorption characteristics of the PET-derived hydrogel and other reported amine-functionalized adsorbents are compared in Table 2. Various types of hydrogels containing amino groups have been designed and studied, including modified nanoporous materials, nanoparticles, and hydrogels. PET-derived hydrogel shows superior adsorption characteristics compared to most reported NH_2_-functionalized adsorbents. Such remarkable adsorption capacity toward NH_2_ is obviously due to the high density of functional amine and amino groups in the hydrogel. Table 2 also shows that the adsorption of Cu^2+^ by most of the studied adsorbents is best fitted with the Langmuir adsorption isotherm, which agrees with the results of the present study. It should be noted that while NH_2_ functional groups play a primary role in the adsorption of Cu^2+^, adsorbents for Cu^2+^ containing only OH groups have been also reported [13], and it is considered that OH groups of the PET-derived hydrogel (Figure 1C) may also contribute to the high Cu^2+^ adsorption capacity of the hydrogel. Similar to the isotherms, the kinetics of Cu^2+^ reported by other studies was second-order adsorption kinetics, which is also consistent with the results obtained for the PET-derived hydrogel.

## 3. Conclusions

Cross-linked hydrogel material prepared from waste PET plastic showed superior characteristics as an adsorbent in the removal of Cu^2+^ as an example of a metal ion of environmental concern. The abundance of NH_2_ ligand groups in the hydrogel determines superior adsorption characteristics of the hydrogel that was demonstrated for Cu^2+^, but potentially applicable to a broad range of other transition metal ions. The proposed hydrogel adsorbent is a sustainable material that simultaneously addresses two environmental issues: waste utilization and pollutant removal. Optimization of the hydrogel preparation method, including minimization of the consumption of chemicals for PET depolymerization and oligomer cross-linking, is important to further reduce the carbon footprint of such waste-plastic-derived materials and enhance their practicality.

## 4. Materials and Methods

### 4.1. Materials

PET was obtained directly from waste soft drink bottles. Diethylenetriamine (DETA) was purchased from Kishida Chemical Co., Ltd. (Osaka, Japan). Ethylene glycol diglycidyl ether (EGDE) was purchased from Tokyo Chemical Industry Co., Ltd. (Tokyo, Japan). The 99.9% dimethyl sulfoxide-d_6_ (DMSO-d_6_), and acetone were purchased from Kanto Chemical Co., Inc. (Tokyo, Japan). Copper (II) nitrate trihydrate (Cu(NO_3_)_2_·3H_2_O) was purchased from Fujifilm Wako Pure Chemical Corporation (Osaka, Japan). Milli-Q water purified by the Purelab Chorus 1 Life Science apparatus was used in all experiments.

### 4.2. Methods

UV–Vis spectroscopy. The UV-vis spectra of aqueous solutions of Cu^2+^ were recorded on a Jasco V-630 spectrophotometer (Japan) in 1 mL quartz cells with an optical path of 1 cm at room temperature.

Fourier Transform Infrared Spectroscopy (FTIR). Aminolysis product of PD and freeze-dried hydrogels were thoroughly mixed with KBr and compressed to form a thin disc, respectively, which were next scanned in a wavenumber range between 4000 cm^−1^ and 400 cm^−1^ using an FTIR spectrometer FTIR-460 (Jasco, Tokyo, Japan) at room temperature.

NMR spectroscopy. PET aminolysis product of PD (ca. 3 mg) and DMSO-d_6_ solution (0.55 mL) were added into an NMR tube. The ^1^H NMR spectrum was measured using a JNM-ECA500 instrument (JEOL, Tokyo, Japan). The chemical shifts in ^1^H NMR were recorded in parts per million (ppm, δ) relative to the solvent resonance as an internal standard (DMSO-d_6_: δ = 2.50 ppm).

X-ray Photoelectron Spectroscopy (XPS). XPS measurements were recorded using a Thermo ESCALAB250Xi X-ray photoelectron spectrometer (Thermo Fisher Scientific, Cambridge, UK), with an analyser pass energy of 200.0 eV. Data acquisition and processing including XPS spectrum fitting were performed using Advantage software (Thermo Fisher Scientific, Cambridge, UK).

Scanning Electron Microscopy (SEM). The SEM observations of the freeze-dried hydrogels were performed at room temperature on a JSM-6610 microscope (JEOL, Japan) equipped with energy-dispersive spectroscopy (EDS) at an acceleration voltage of 15 kV.

### 4.3. Sample Preparation

Preparation of PET-derived hydrogels. The aminolysis depolymerization of PET was performed in the microwave reactor (Monowave 400, Anton Paar) under autogenous pressure. Of the PET, 1 g and 3 mL of DETA were added into the microwave reactor, and the reaction was carried out at 200 °C for ca. 1 min. Then, 0.2 g of the resulting aminolysis product was transferred to 2 mL of Milli-Q water and heated to 100 °C until complete dissolution. Consequently, 352 µL of EDGE was added at 50 °C and 250 rpm stirring in a petri dish. After 20 ± 5 min, the solution became viscous, and the stirring was stopped. Finally, the reaction mixture was incubated at 50 °C for 10 min and at room temperature overnight to ensure the completeness of gelation. The hydrogel was washed with Milli-Q water and stored in Milli-Q water. The dry samples of the hydrogels were prepared through the lyophilizing of the hydrogel using an Eyela FDU-1200 freeze-drier (Japan).

The swelling ratio of hydrogel was calculated using Equation (1).

(1) $$S=\frac{m_{wet}-m_{dry}}{m_{dry}}$$

in which $m_{wet}$ and $m_{dry}$ are the weights of the swollen and lyophilized hydrogels, respectively.

Adsorption of Cu^2+^ by the hydrogel. Generally, a piece of hydrogel was placed into Cu^2+^ solutions of different concentrations. The decrease in Cu^2+^ concentration in the solution above the hydrogel was measured via UV-vis spectroscopy. To investigate the kinetic behaviour, ca. 1 g of hydrogel was used for the adsorption of 15 mL of 127 mg/L Cu^2+^ solution at room temperature. The Cu^2+^ concentrations were measured via UV-vis spectroscopy at different time intervals. The impact of the initial concentration of Cu^2+^ on the adsorption capacity was studied using ca. 0.15 g of hydrogel for 4.5 mL of the Cu^2+^ solution at concentrations ranging from 63 to 1270 mg/L. To study the effect of the pH of the Cu^2+^ solution on the adsorption capacity, ca. 0.13 g of the hydrogel was used for the adsorption of 4.5 mL of a 127 mg/L Cu^2+^ solution under a pH in the range of 2–6 at room temperature.

The adsorption capacity ($Q_{e}$) was calculated using the following Formulas (2):

(2) $$Q_{e}=\frac{\left(C_{0}-C_{t}\right)V}{m}$$

In which $C_{0}$ (mg/L) is the initial concentration of the Cu^2+^ solution, $C_{t}$ (mg/L) is the concentration of the Cu^2+^ solution at time t (min), *V* (L) is the Cu^2+^ solution volume, and *m* (g) is the mass of the adsorbent.

The fitting of Cu^2+^ adsorption data by pseudo-first-order and pseudo-second-order kinetic models was performed using Formulas (3) and (4), respectively.

(3) $$Q(t)=Q_{e}(1-e^{k_{1}t})$$

(4) $$Q(t)=\frac{Q_{e}^{2}k_{2}t}{1+Q(t)k_{2}t}$$

in which *Q*(*t*) (mg/g) is the amount of adsorbed Cu^2+^ at time *t* (min), $Q_{e}$ (mg/g) is its value at equilibrium, $k_{1}$ (min^−1^) and $k_{2}$ (g/mg·min) are the pseudo-first-order and pseudo-second-order model kinetic rate constants, respectively.

The fitting of Cu^2+^ adsorption data using Langmuir and Freundlich isotherm models was performed using Formulas (5) and (6), respectively.

(5) $$Q_{e}=\frac{K_{L}Q_{m}C_{e}}{1+K_{L}C_{e}}$$

(6) $$Q_{e}=K_{F}C_{e}^{1/n}$$

in which $C_{e}$ (mg/L) is the concentration of Cu^2+^ at the equilibrium, $Q_{m}$ (mg/g) is the maximum amount of the adsorbed Cu^2+^, $K_{L}$ (L/mg) is the Langmuir isotherm constant, $K_{F}$ (mg/g) is the Freundlich isotherm constant, and $\frac{1}{n}$ is the degree of non-linearity of the Freundlich isotherm model.

Desorption of Cu^2+^ by the hydrogel. The desorption experiments were carried out using 15 mL of 0.1 M EDTA solution. At first, ca. 1 g of the hydrogel was used to adsorb 15 mL of a 127 mg/L Cu^2+^ solution at room temperature. Then, the Cu-contained hydrogel was treated with 0.1 M of EDTA solution. The concentration of Cu^2+^ in the solution was measured via UV-vis spectroscopy. The subsequent Cu^2+^ adsorption experiments were carried out with the regenerated hydrogel and the above process was repeated three times.

## Figures and Tables

**Table 1:** Fitting parameters of the kinetics and adsorption isotherms.

Kinetic Model	Pseudo-First Order			Pseudo-Second Order		
	*Q*_e (cal)_ (mg/g)	*k*_1_ (min^−1^)	*R* ^2^	*Q*_e (cal)_ (mg/g)	*k*_2_ (g/mg·min)	*R* ^2^
	44	0.0067	0.9873	58	1.02 × 10^−4^	0.9909
Isotherm model	Langmuir			Freundlich		
	*Q*_m (cal)_ (mg/g)	*K*_L_ (L/mg)	*R* ^2^	*K*_F_ (mg/g)	n	*R* ^2^
	248	0.0054	0.9930	18	2.8	0.9488

**Table 2:** Comparison of Cu^2+^ adsorption capacities of the PET-derived hydrogel with other amine-containing adsorbents at room temperatures and a neutral pH.

Material	Adsorption Capacity/mg/g	Kinetics Model	Adsorption Model	Ref.
Amine-functionalized mesoporous C_3_N_4_	200	Pseudo-second	Langmuir	[22]
Amine-functionalized biochar	74.5	Pseudo-second	Sips	[23]
Amine-functionalized carbon nanotubes	26.4	Pseudo-first	Langmuir	[24]
Amine-functionalized SBA-15	34.3	-	Sips	[25]
Amine-functionalized magnetic particles	10.4	Pseudo-second	Langmuir	[26]
Amine-functionalized mesoporous silica	52.7	Pseudo-second	Langmuir	[27]
Chitosan hydrogel beads	163.9	-	Langmuir	[28]
Polyethylenimine-functionalized hydrogel	40.0	Pseudo-second	Langmuir	[29]
Poly(amidehydrazide) hydrogel	85	-	Sips	[30]
This study	213	Pseudo-second	Langmuir	-

## Data Availability

Data is contained within the article or Supplementary Material.

## Supplementary Materials

The following supporting information can be downloaded at: https://www.mdpi.com/article/10.3390/gels9110874/s1, Figure S1: Dependences of the microwave power and reaction mixture temperature on the reaction time.
