# Supplementary material for: Copper Ion Removal Using a Waste-Plastic-Derived Hydrogel Adsorbent Prepared via Microwave-Assisted PET Aminolysis
Source: Gels. 2023 Nov 3;9(11):874. doi: 10.3390/gels9110874 (PMC10670419; doi:10.3390/gels9110874)
Supplement: Supplementary file 1 [file gels-09-00874-s001.zip › gels-2687432-supplementary.pdf]

Supplementary Material

Copper Ion Removal Using a Waste-Plastic-Derived Hydrogel Adsorbent Prepared via Microwave-Assisted PET Aminolysis

Kayee Chan,[1,*] Masami Kawai,[2,†] Mina Yamake,[3,†] Anatoly Zinchenko[1,*]

[1] Graduate School of Environmental Studies, Nagoya University, Furo-cho, Chikusa-ku, Nagoya 464-8601, Japan
[2] Gifu High School, 3-1, Onawaba, Gifu 500-8889, Japan
[3] Gifu Kita High School, 1841-11, Noritake, Gifu 502-0931, Japan

* Correspondence: chan.kayee.v2@s.mail.nagoya-u.ac.jp (K.C.); zinchenko@urban.env.nagoya-u.ac.jp (A.Z.);
Tel.: +81-52-789-7045 (K.C.); +81-52-789-4771 (A.Z.)

† These authors contributed equally to this work.

1. Experimental conditions of microwave-assisted depolymerization of PET

The aminolysis of PET was performed in the microwave reactor (Monowave 400, Anton Paar) under autogenous pressure. The reaction was carried out at 200 °C for *ca.* 1 minute **(Figure S1)**.

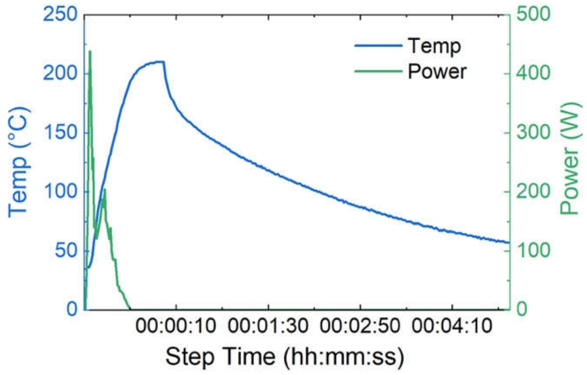

Figure S1. Dependences of the microwave power and reaction mixture temperature on the reaction time.
